# Network pharmacology and Mendelian randomization analysis of Xiao Bi decoction in treating psoriasis

**DOI:** 10.1097/MD.0000000000044173

**Published:** 2025-08-29

**Authors:** Wentao Hu, Yifang Jiang, Jie Wang, Min Jia, Chunlan Wu, Changhui Wen

**Affiliations:** a Guizhou University of Traditional Chinese Medicine, Guiyang, Guizhou, China; b Department of Endocrinology, The First Affiliated Hospital of Guizhou University of Traditional Chinese Medicine, Guiyang, Guizhou, China; c Department of Dermatology, The First Affiliated Hospital of Guizhou University of Traditional Chinese Medicine, Guiyang, Guizhou, China; d Department of Hospital Sense, First Affiliated Hospital of Guizhou University of Traditional Chinese Medicine, Guiyang, Guizhou, China.

**Keywords:** Mendelian randomization (MR), molecular docking, network pharmacology, psoriasis, traditional Chinese medicine, Xiao Bi Decoction (XBT)

## Abstract

Psoriasis is a chronic autoimmune disease marked by excessive keratinocyte growth and immune issues. Xiao Bi decoction (XBT), a traditional Chinese formula, has shown effective for treating psoriasis. This study integrated network pharmacology and Mendelian randomization analysis to explore the therapeutic mechanism of XBT and validate causal relationships between its active components, potential targets, and psoriasis outcomes. The results showed that XBT contains 171 active components, of which the top 5 active components with the highest degree values were quercetin, dihydrobaicalin _qt, Pyrethrin II, Kinobeon A, and Baicalein; the main core targets of XBT were tumor necrosis factor, interleukin (IL)-6, glyceraldehyde-3-phosphate dehydrogenase, AKT1, and IL-1B. These core components and core targets can be better molecular docking, and Mendelian randomization analysis showed that there is a causal relationship between the reduced level of IL-6 and the increased risk of psoriasis. These results provide novel insights into the molecular mechanisms and scientific validation of XBT’s use in psoriasis.

## 1. Introduction

Psoriasis is a chronic immune-related skin disease with recurring lesions, common in dermatology. However, the etiology of psoriasis remains unclear. Owing to its aesthetic nature, a change in appearance often causes a greater physical and mental burden on the patient. Compared with other chronic diseases, such as heart failure, psoriasis is second only to depression and chronic lung disease in terms of its impact on psychological quality of life.^[1]^ The adverse physical effects of psoriasis are incompletely understood, and are currently thought to be related to common symptoms such as itching, pain, burning, and bleeding, as well as psychological burden due to changes in image.^[2]^ Some patients with psoriasis are at significantly higher risk of other complications, including psoriatic arthritis, metabolic syndrome, cardiovascular disease, anxiety, depression, nonalcoholic fatty liver disease, Crohn disease, and lymphoma.^[3]^

Currently, there are many treatment options, and generally speaking, they can be treated separately according to the condition: for mild psoriasis, topical corticosteroids, vitamin D analogs, calmodulin neural phosphatase inhibitors, stratum corneum separators, and targeted phototherapy. For moderate to severe psoriasis, it is currently recommended to use a combination of multiple interventions, as well as injectable molecularly targeted drugs, oral systemic therapeutic drugs, and phototherapy.^[4]^ The 4 biomolecule-targeted agents used for the treatment of moderate to severe plaque psoriasis are tumor necrosis factor (TNF) inhibitors, interleukin (IL)-12/23 inhibitors, IL-17 inhibitors, and IL-23 inhibitors.^[5]^ However, patients with psoriasis treated with biologics, such as nasopharyngitis and upper respiratory tract infections, have a slightly higher incidence of adverse effects.^[6]^ Therefore, safer and more effective treatments for psoriasis are required.

In Chinese medicine, the treatment of psoriasis with Xiao Bi decoction (XBT) has been clinically applied for more than 10 years, and a large number of studies have confirmed that patients with psoriasis have exact efficacy and good safety after taking XBT. Wen et al^[7]^ found that XBT can reduce the MED value of guinea pigs, and at the same time, it can increase the sensitivity of skin lesions to a wavelength of 308 nm excimer light, as well as the amount of absorption of the lesions. The results of the study of psoriasis-like lesions in a rat model showed that the results of the study on rat psoriasis-like skin lesion model by Hu et al^[8]^ showed that XBT could achieve the same therapeutic effect as that of acitretin capsule. However, the specific mechanism of psoriasis treatment by XBT therapy needs to be further investigated. Due to the complexity of Chinese medicine compounding, the complexity of components, multiple links of action, difficulty to quantify, and other characteristics, compounding and other research is often a certain degree of difficulty. Network pharmacology elucidates drug mechanisms through multi-gene/target/pathway interaction networks.^[9]^ Another key tool, molecular docking, is widely used in drug discovery to estimate the binding structure of small molecule ligands to their corresponding target binding sites.^[10]^ In addition, numerous factors can cause or trigger psoriasis. In many cases it is not possible to determine exactly which factor directly causes or triggers it, Mendelian randomization (MR) is an approach that uses single nucleotide polymorphisms (SNPs) as instrumental variables to assess the causal relationship between exposure factors and disease.^[11]^

Therefore, in this study, first, network pharmacology identified XBT’s core targets against psoriasis, validated through molecular docking analysis, and finally, MR using the core targets of XBT as the exposure factors and psoriasis as the endpoints were combined to reveal the molecular mechanism of XBT for the treatment of psoriasis.

## 2. Methods

### 2.1. Collection of active compounds and targets

Based on the Traditional Chinese Medicine Systems Pharmacology Database and Analysis Platform (TCMSP, https://www.tcmsp-e.com/index.php) database,^[12]^ we reviewed the literature (CNKI and PubMed) to search for all the chemical constituents contained in each herb of XBT (the search period was from January 2018–October 2024). XBT is composed of 13 Chinese herbs, and for the Chinese medicines that could be retrieved by TCMSP, active compounds were filtered based on oral bioavailability (OB ≥ 30%) and drug-likeness (DL ≥ 0.18).^[13]^ Components not included in the TCMSP database were filtered using the SwissADME (http://www.swissadme.ch/) database^[14]^ based on “high” OB and DL with at least 3 “yes” items.^[15]^

### 2.2. Screening of drug targets

All screened active ingredients were predicted for targets using SwissTargetPrediction (http://www.swisstargetprediction.ch/).^[16]^ Target matching was performed using the Universal Protein database (http://www.uniprot.org/).^[17]^

### 2.3. Psoriasis-related genes

We searched the Genecards (https://www.genecards.org/),^[18]^ Online Mendelian Inheritance in Man (https://omim.org/#),^[19]^ and the Therapeutic Target Database (http://db.idrblab.net/ttd/) were searched to obtain psoriasis-related targets,^[20]^ and the targets were combined and duplicates removed.

### 2.4. Construction of a “drug–component–target” network

The active ingredients and targets were collated using Excel software, and the data were imported into Cytoscape 3.9.1 to draw a “drug–component target” network, with diseases, drugs, components, and related targets as nodes and their interrelationships as edges. Topological analyses were performed to identify the core components of the network (mainly in terms of the degree values).

### 2.5. Construction of protein–protein interaction (PPI) network

Drug and disease targets were taken as intersections to obtain common targets. The intersection targets were analyzed by PPI (https://string-db.org).^[21]^ The PPI network was constructed using the STRING database (https://www.string-db.org/) through the following workflow: first, putative target proteins were imported into the platform with species specification set to *Homo sapiens*. Subsequently, the confidence threshold for interaction scoring was established at 0.4 (medium confidence) based on experimental and computational evidence. To enhance network visualization clarity, nodes lacking physical/functional associations were systematically excluded by activating the “Hide disconnected nodes” option under Network Display Parameters, and then the analytical data obtained from the String network were imported into Cytoscape 3.9.1 software for PPI network visualization and analyzed by the CentiScaPe 2.2 plug-in was used to calculate the degree, betweenness centrality, and closeness centrality of each active ingredient, and according to the 3 topological parameters were greater than the threshold value, we obtained the core gene target of the XBT.^[22]^

### 2.6. Gene ontology (GO) and Kyoto encyclopedia of genes and genomes (KEGG) pathway enrichment analyses

To validate the association of intersecting genes with psoriasis, we used the “clusterProfiler” package (Jinan University Guangzhou, China) to enrich for intersecting targets in *H sapiens*, including biological processes, cellular components, and molecular functions. Statistical significance was defined by dual-threshold criteria requiring both an uncorrected *P* value < .05 and a Benjamini–Hochberg false discovery rate (FDR) < 0.05. The resultant data were visualized using the ggplot2 package for comprehensive graphical representation, complemented by functional enrichment analysis performed through the GOplot toolkit to elucidate biological pathway associations.

### 2.7. Molecular docking

Molecular docking was performed between XBT active compounds from the “drug–component–target” network and the key target proteins screened in the construction of the PPI network. The docking software AutoDock Tools 1.5.6 and Autodock vina 1.2.0 (developed and maintained by The Scripps Research Institute, La Jolla) were used to dock the active ingredient of the XBT and the target proteins,^[23]^ to obtain their lowest binding energies, and the molecular docking results were visualized and analyzed using PyMOL (3.1.3) software.

### 2.8. MR analysis between feature genes and psoriasis

We performed a two-sample MR analysis to explore causal associations between core genes and psoriasis risk by defining SNPs as instrumental variables. We obtained SNPs for core genes as exposure factors and psoriasis as outcome factors from the Integrated Epidemiology Unit database (https://gwas.mrcieu.ac.uk/). The instrument selection protocol followed these steps: initial genome-wide significance threshold was set at *P* < 5 × 10⁻⁸ (conventional threshold for genome-wide association studies), which yielded insufficient instrument density (n < 10) for robust MR analysis. To optimize the instrument strength-sample size balance, we empirically adopted a more inclusive threshold of *P* < 5 × 10⁻⁶ based on established pharmacogenomic criteria, which provided adequate genetic instruments while maintaining biological plausibility. All candidate SNPs underwent clumping procedures using PLINK 2.0 with stringent linkage disequilibrium parameters: physical distance threshold of 5000 kilobases and pairwise *r*² < 0.1. Instrument strength validation was performed through *F*-statistic calculation, where variants with *F* > 10 (indicating < 10% weak instrument bias) were retained as valid instrumental variables. This multistep filtering strategy effectively balanced type I error control and statistical power in subsequent causal inference analyses. MR analyses were performed using the ‘TwoSampleMR’ package (developed and maintained by Medical Research Council Integrative Epidemiology Unit [ MRC-IE ], Bristol, UK), and the instrument selection protocol was rigorously implemented through the following steps: initial genome-wide significance threshold was set at *P* < 5 × 10⁻⁸ (conventional threshold for genome-wide association studies), which yielded insufficient instrument density (n < 10) for robust MR analysis. To optimize the instrument strength–sample size balance, we empirically adopted a more inclusive threshold of *P* < 5 × 10⁻⁶ based on established pharmacogenomic criteria, which provided adequate genetic instruments while maintaining biological plausibility. All candidate SNPs underwent clumping procedures using PLINK 2.0 with stringent linkage disequilibrium parameters: physical distance threshold of 5000 kilobases and pairwise *r*² < 0.1. Instrument strength validation was performed through *F*-statistic calculation, where variants with *F* > 10 (indicating < 10% weak instrument bias) were retained as valid instrumental variables. This multistep filtering strategy effectively balanced type I error control and statistical power in subsequent causal inference analyses. The inverse variance weighted (IVW) method was used to assess the relationship between the expression levels of the key target genes and the risk of psoriasis. IVW assumes the validity of all genetic instruments and thedirectly proportional relationship between outcome and exposure. In this method, the Wald ratio is calculated for each SNP to determine its effect on the outcome, and then are fitted using the inverse of the outcome variance (the square of the standard error) as the weight.^[24]^ Additionally, a sensitivity analysis was performed using the MR-Egger method to ensure the robustness of our findings. We also applied Cochran *Q* statistic to test for heterogeneity, where *P* < .05, indicating heterogeneity in the IVW results. Potential horizontal pleiotropy was assessed using MR-Egger regression and MR-PRESSO analyses, where *P* > .05, indicated the presence of horizontal pleiotropy in IVW results (https://gwas.mrcieu.ac.uk/).^[25,26]^

## 3. Results

### 3.1. Identification of active components and targets

According to the ADME parameters (OB ≥ 30% and DL ≥ 0.18), the 13 herbs in this formula contained at least 183 active ingredients, of which 12 were duplicates. There were 36 types of huangqin, 23 types of jinyinhua, 23 types of lianqiao, 11 types of pugongying, 10 types of jihuadiding, 2 types of dihuang, 2 types of wushaoshe, 6 types of fengfang, 4 types of shuizhi, 23 types of taoren, 22 types of Honghua, 13 types of banxia, and 7 types of guizhi (see Table S1, Supplemental Digital Content, https://links.lww.com/MD/P800 for details).

### 3.2. Construction and analysis of relevant targets and the “drug–component–target” network

**Figure 1.**
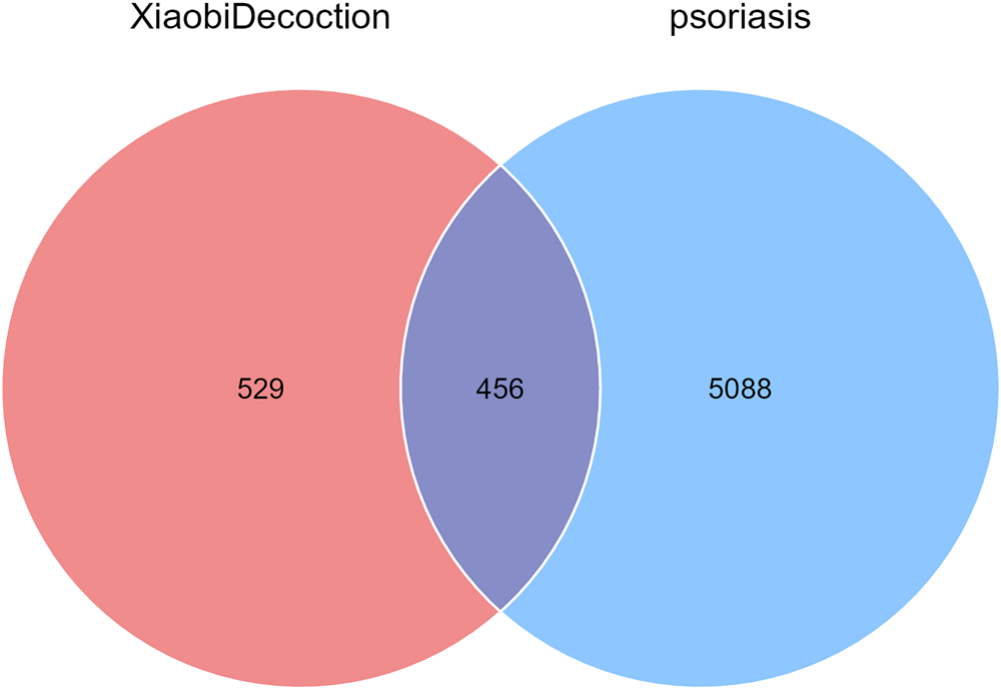
Venn diagram of drug–disease target intersection.

**Figure 2.**
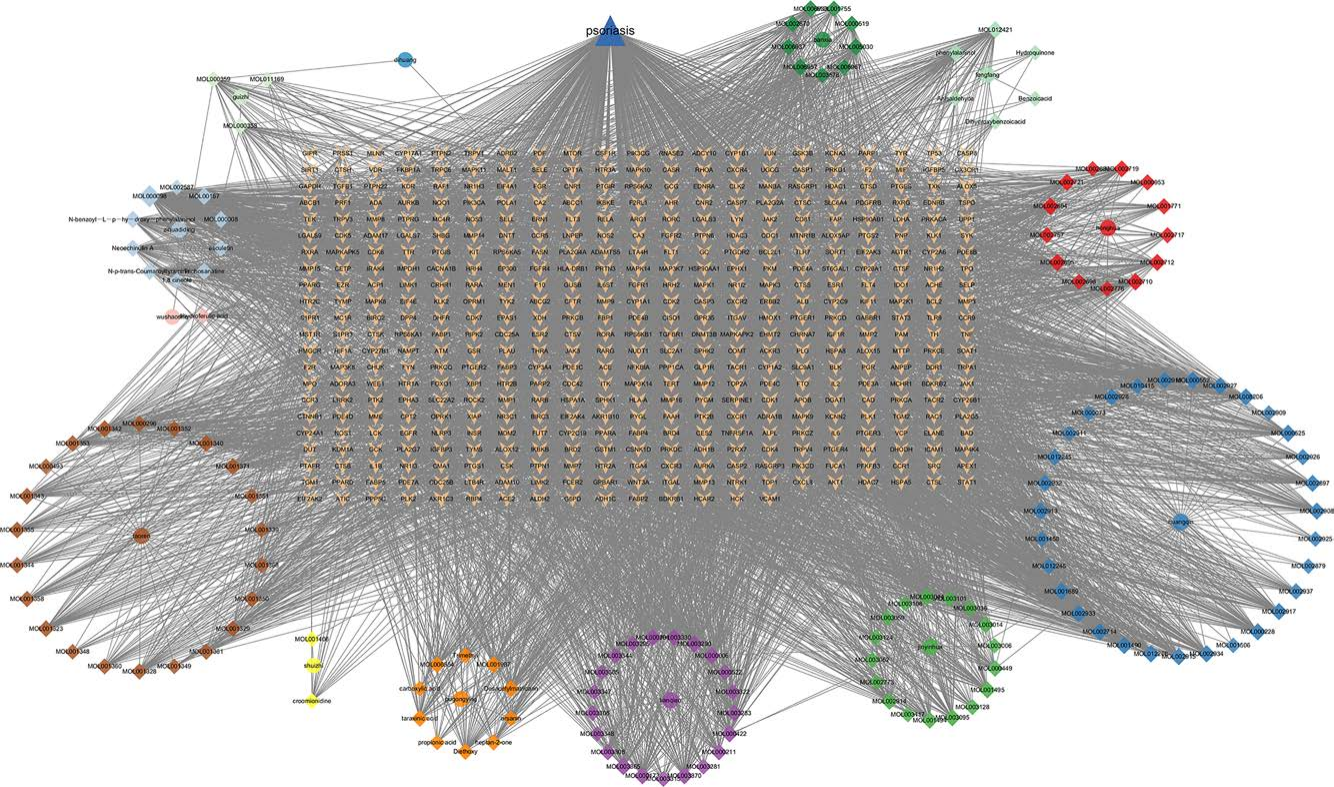
“Drug–component–target” network. Thirteen circles represent 13 types of TCM, each color represents 1 type of drug, the prisms correspond to each component of the colored TCM, and the size and transparency of the various shapes are related to the corresponding degree values. TCM = traditional Chinese medicine.

A total of 985 possible targets of the active chemical components of the XBT compound prescriptions were obtained through screening. The total number of possible psoriasis-related disease targets was 5544. The drug and disease targets were intersected to obtain 456 common targets (Fig. 1), representing 46.29% (456/985) of the herbal targets and 8.22% (456/5544) of the disease targets. The “drug–component–target” network is shown in Figure 2, and the network analysis results showed that there were 615 nodes and 5229 edges. Among them, quercetin, dihydrobaicalin _qt, and Pyrethrin II had the highest degree values, and Kinobeon A baicalein had the same degree value after the above 3 components. Topological analysis revealed elevated node degrees in the drug–compound–target network, indicating enhanced polypharmacological interactions. Molecular validation demonstrated quercetin’s preferential binding to PTGS2, suggesting XBT exerts anti-psoriatic effects through PTGS2-mediated COX-2/PGE2 pathway inhibition and CA2-regulated epidermal barrier restoration.

### 3.3. PPI network construction and analysis

**Figure 3.**
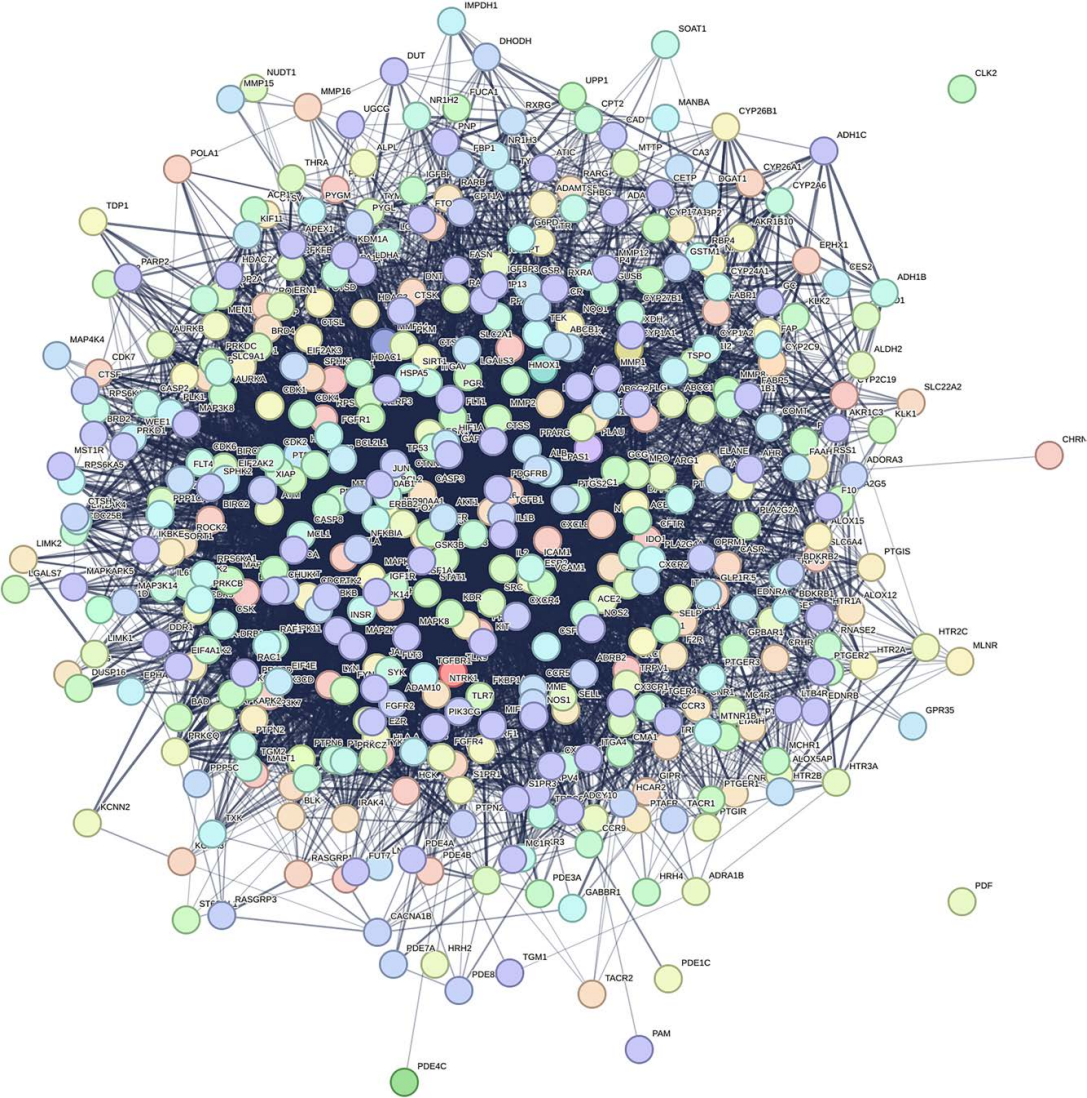
Drug–disease intersection target protein–protein interactions map.

**Figure 4.**
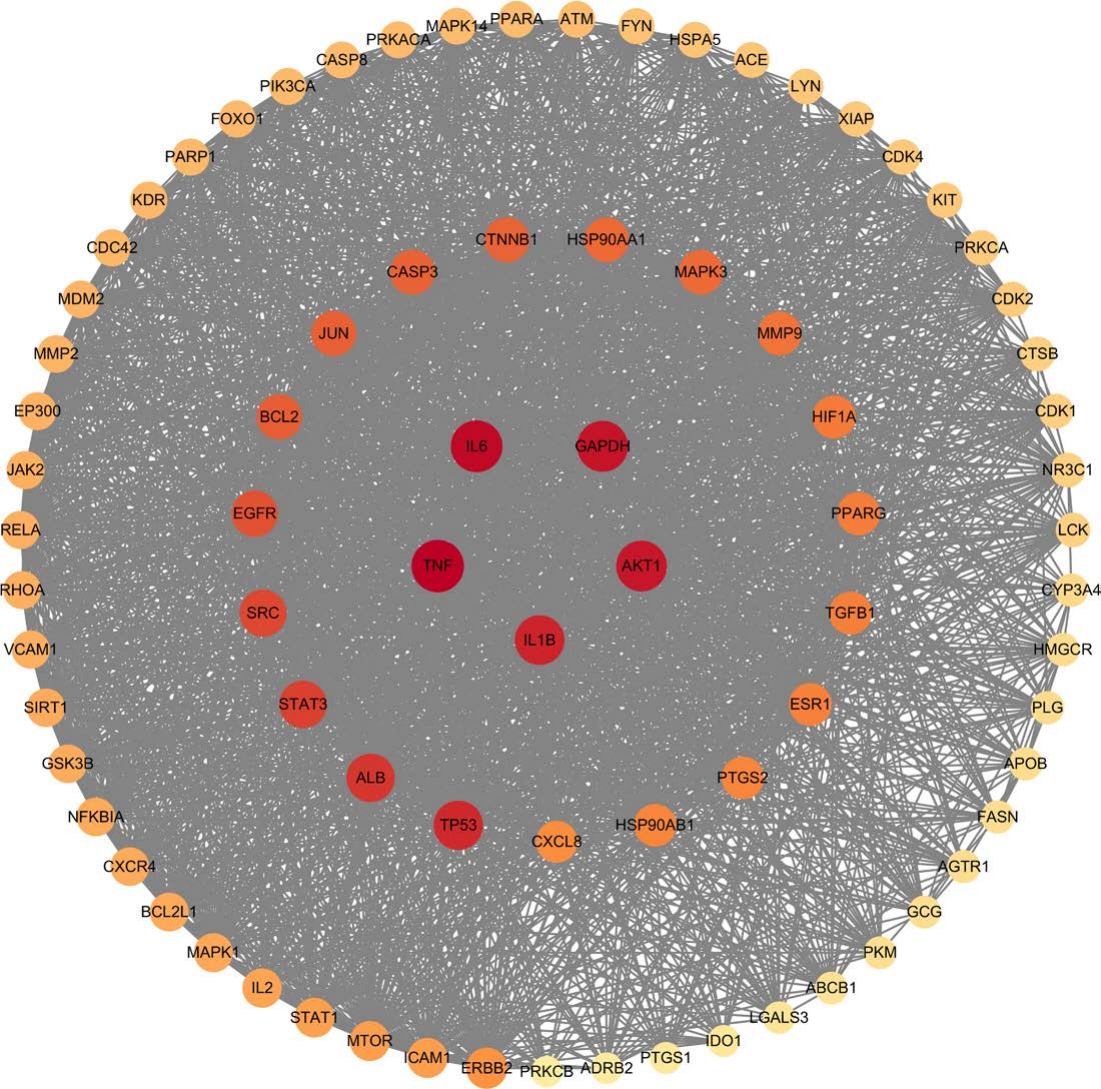
Core genes. Each circle represents each intersection gene, and the closer the circle color is to red, the larger the degree value. GAPDH = glyceraldehyde-3-phosphate dehydrogenase, IL = interleukin, TNF-α = tumor necrosis factor-α.

String analysis of the protein interactions for the intersecting targets is shown in Figure 3. CentiScaPe 2.2 analysis revealed 324 genes exceeding thresholds for degree, betweenness centrality, and closeness centrality, of which, TNF, IL-6, glyceraldehyde-3-phosphate dehydrogenase (GAPDH), AKT1, and IL1B had the highest and decreasing degree values, and the core target PPI network is shown in Figure 4.

### 3.4. GO and KEGG enrichment analysis

**Figure 5.**
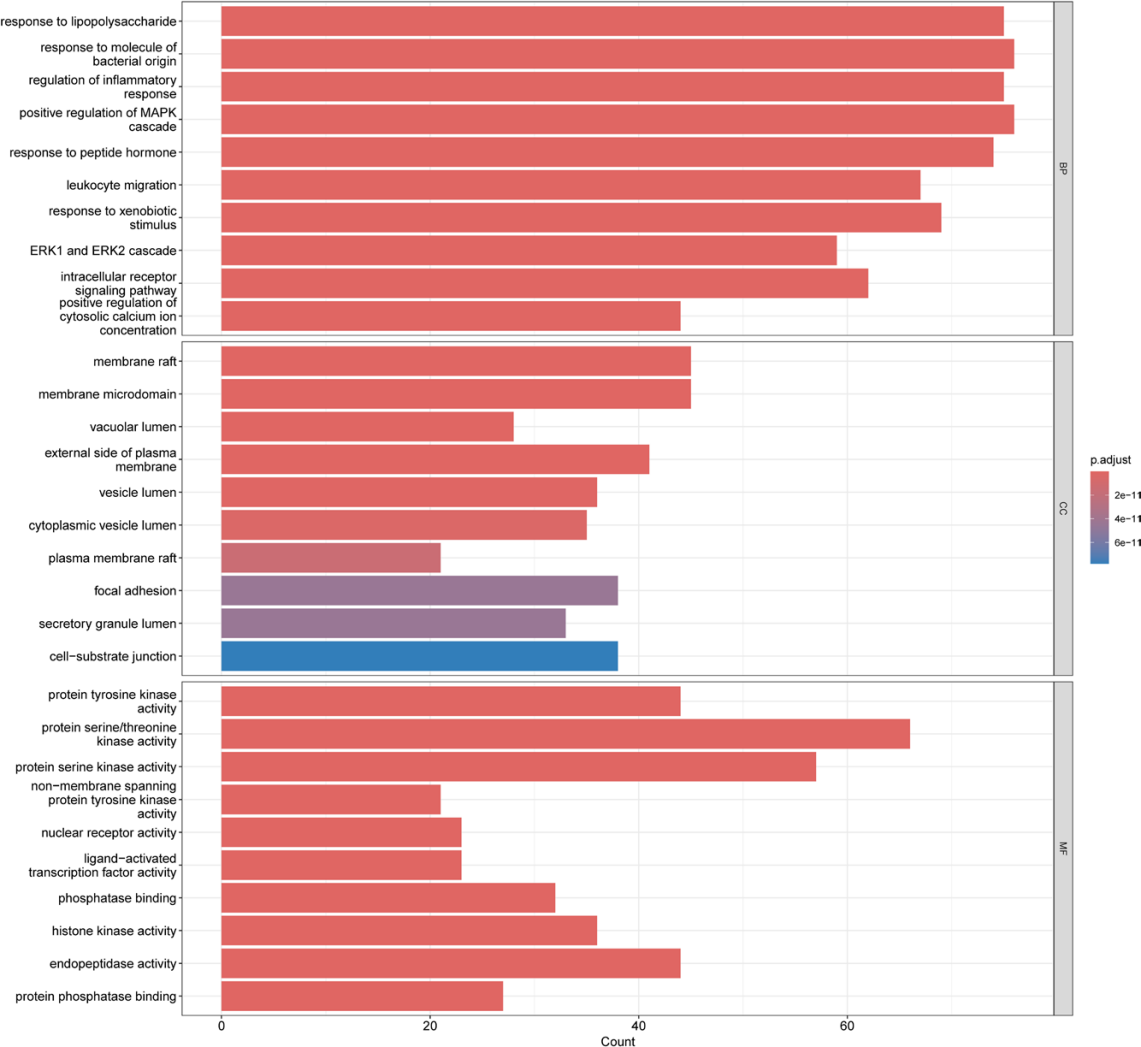
Histogram of 30 vitally enriched go terms, including the top 10 BPs, CCs, and MFs. GO analysis was conducted using clusterProfiler. BPs = biological processes, CCs = cellular components, GO = gene ontology, MFs = molecular functions.

**Figure 6.**
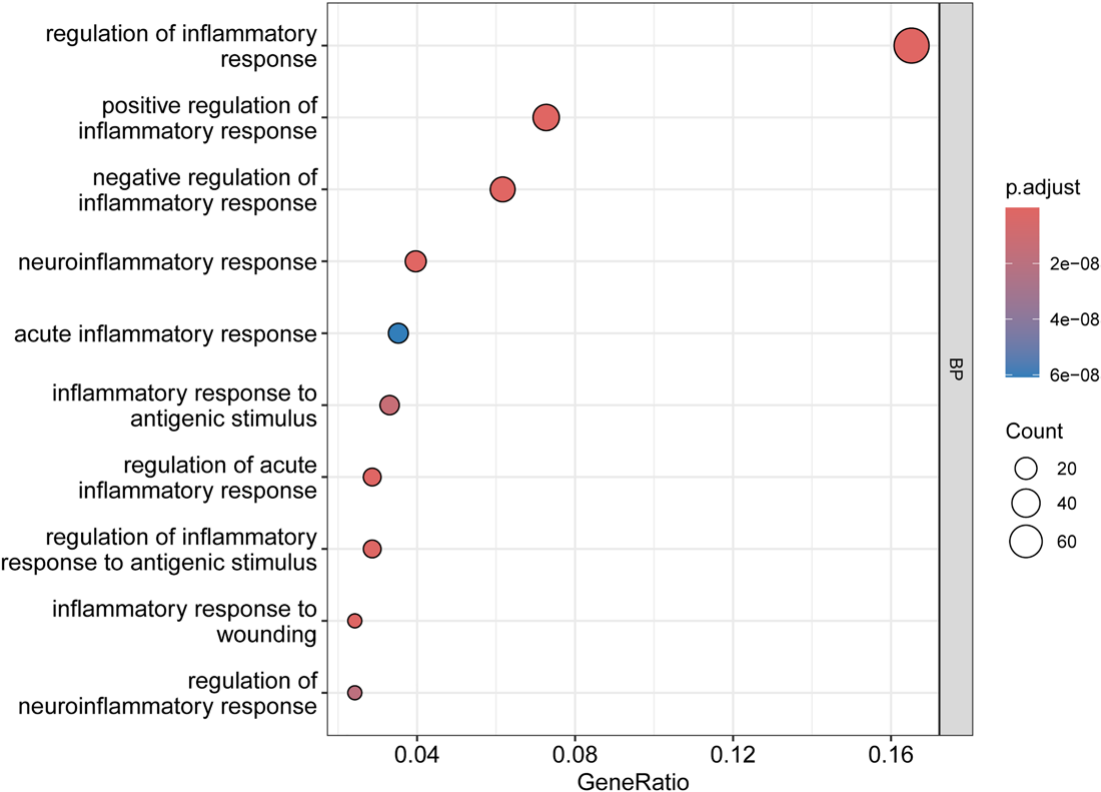
Bubble diagrams of the enriched GO pathways, including 10 terms belonging to inflammatory pathway processing. GO = gene ontology.

**Figure 7.**
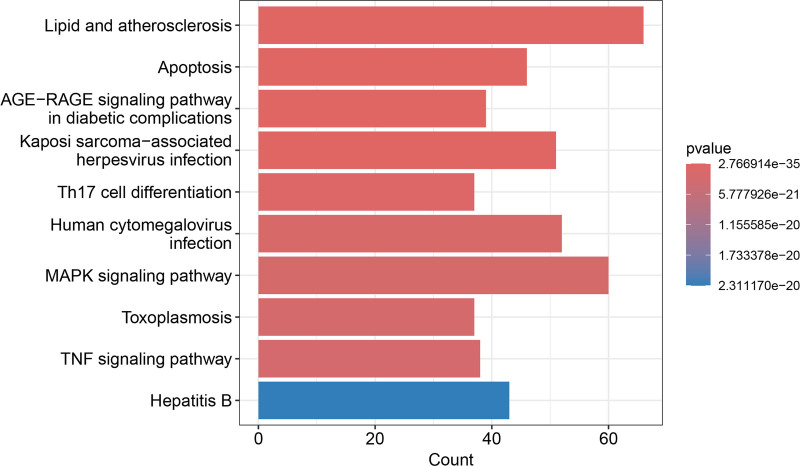
Histogram of KEGG top 10 enrichment pathways. KEGG = Kyoto encyclopedia of genes and genomes.

GO and KEGG pathway enrichment analyses were performed to elucidate the biological functions and pathways associated with these 456 potential targets. Subsequently, the top 10 enriched GO-enriched pathways were selected from the biological process, cellular component, and molecular function categories and are presented in Figure 5. Some of the components of XBT are involved in a variety of signaling pathways, especially in the molecular mechanisms of inflammation. Bubble (Fig. 6) shows 10 relevant inflammatory pathways. In addition, Figure 7 demonstrated the top 10 KEGG-enriched pathways, in which all of these pathways, Th17 cell differentiation, MAPK signaling pathway, and TNF signaling pathway, were all highly associated with psoriasis.

### 3.5. Molecular docking

**Figure 8.**
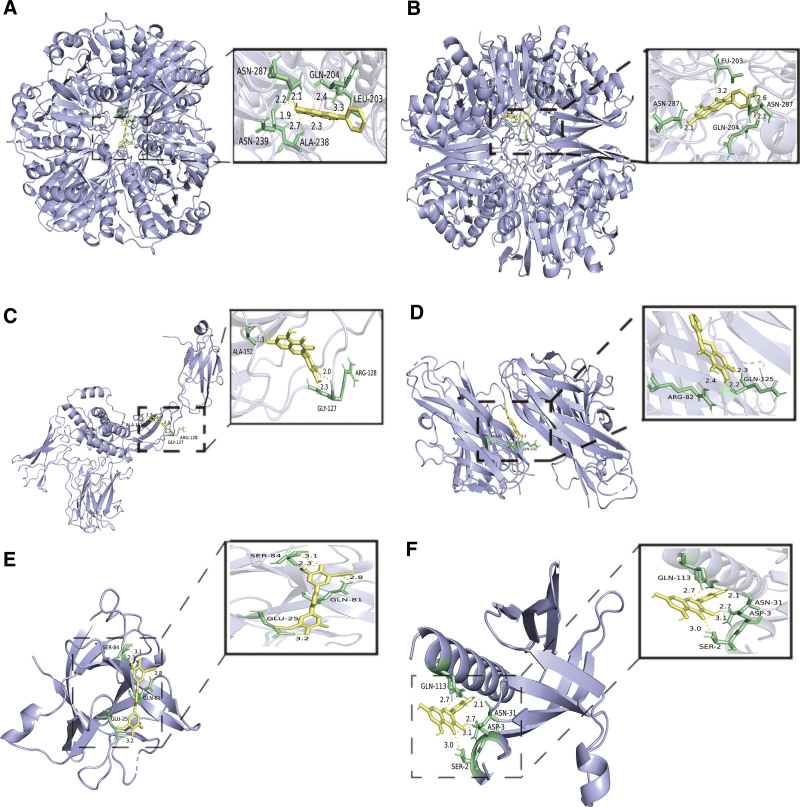
Molecular docking assemblies, (A) baicalein–GAPDH, (B) quercetin–GAPDH, (C) quercetin–IL-6, (D) Dihydrobaicalin_qt–TNF, (E) Kinobeon A–IL-1B, (F) quercetin–AKT1. Bright blue represents macromolecular protein structures (targets), yellow represents small molecule structures (drug ingredients), the yellow dotted line and the numbers next to it represent the hydrogen bonds and bond energies formed, and green represents the residues. GAPDH = glyceraldehyde-3-phosphate dehydrogenase, IL = interleukin, TNF = tumor necrosis factor.

The molecular docking results are shown in Table 1. The docking binding energies of all the core genes and core components of XBT are lower than −5 kcal/mol, suggesting most genes form stable structures with core compounds. We selected some of the pairwise combinations to be shown in Figure 8, and it can be seen that there are hydrogen bonds with each protein residue in each pairwise combination in Figure 8; among these, baicalein binds to the residues of GAPDH ASN-287, ASN-239, ARA-238, and CLN-204 LEU-203 through hydrogen bonds with very strong binding affinity (Fig. 8A), and the docking binding affinity was −8.968 kcal/mol.

**Table 1:** Molecular docking binding energies.

Mol ID	Mol name	Target				
		TNF	IL-6	GAPDH	AKT1	IL-1B
MOL000098	Quercetin	-7.788	-8.029	-8.836	-6.358	-7.087
MOL002913	Dihydrobaicalin_qt	-8.468	-7.554	-8.829	-6.825	-6.782
MOL002710	Pyrethrin II	-7.4	-6.531	-8.033	-5.397	-5.867
MOL002694	Kinobeon A (pubchem name)	-7.27	-6.793	-7.682	-5.986	-5.824
MOL002714	Baicalein	-8.423	-7.399	-8.968	-6.797	-7.038

GAPDH = glyceraldehyde-3-phosphate dehydrogenase, IL = interleukin, TNF = tumor necrosis factor.

### 3.6. Mendelian randomization analysis of core genes and psoriasis

**Figure 9.**
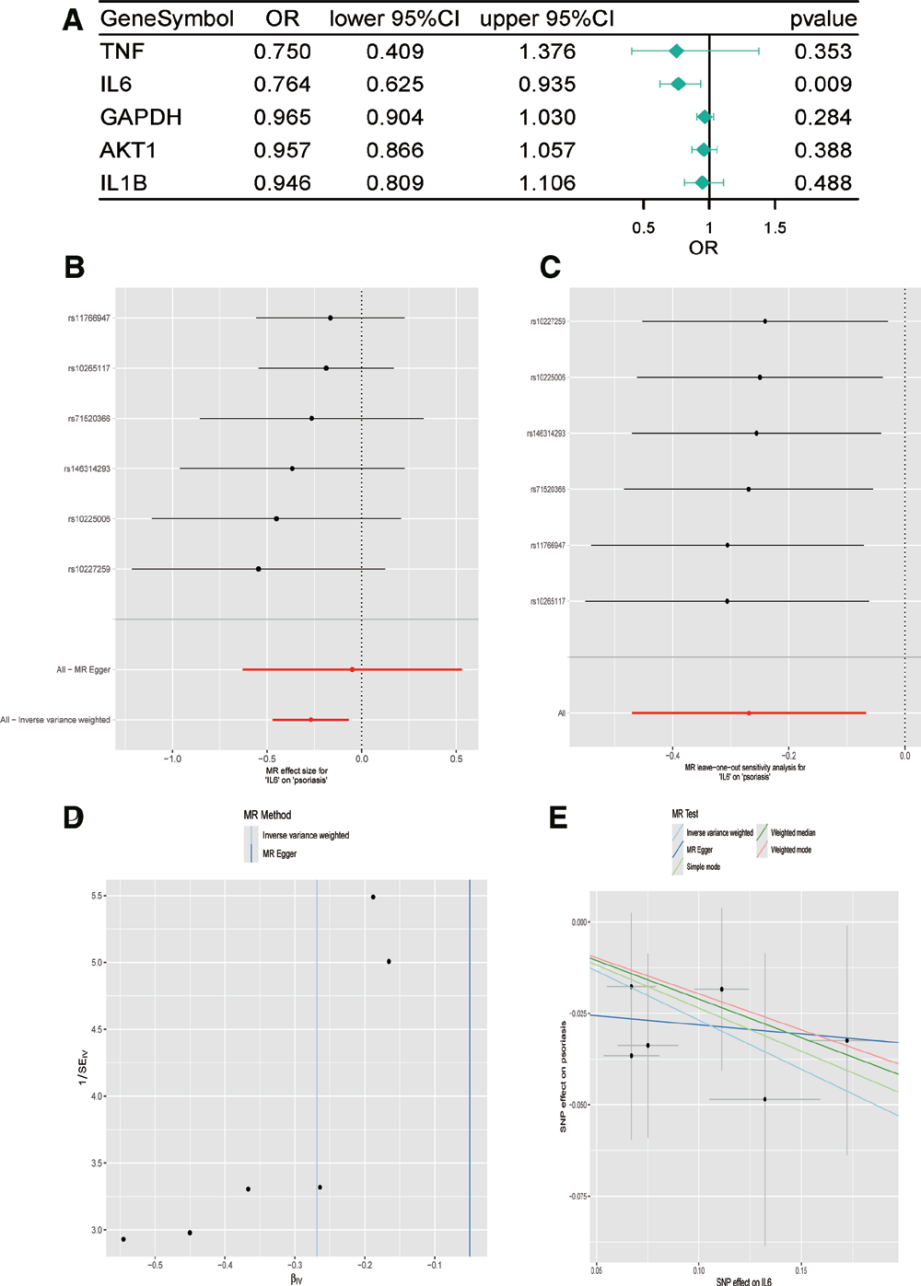
(A) Forest plot of causality between the 5 core genes and psoriasis under the IVW approach. (B) Forest plot of causality of each SNP in IL-6 on DN risk. (C) Leave-one-out plot of IL-1B on DN risk when leaving 1 SNP out. (D) Funnel plot of IL-6 on psoriasis. (E) Scatterplot of causality of IL-6 on psoriasis risk. IL = interleukin, IVW = inverse variance weighting, SNPs = single nucleotide polymorphisms.

We verified the correlation between the 5 core genes and psoriasis using MR analysis, and the results are shown in Figure 9A. The IVW analysis confirmed a significant causal relationship between IL-6 and psoriasis risk (OR = 0.764, *P* = .009); however, the other 4 core targets were not directly correlated with psoriasis. The IVW analysis of IL-6, its causal impact on psoriasis is shown in Figure 9B and C. The funnel plot illustrates a concentrated distribution of IL-6 eQTL effect estimates against inverse standard error in both IVW and MR-Egger analyses. The symmetrical clustering around IVW’s central estimate with minimal dispersion suggests low heterogeneity and absence of significant outliers. MR-Egger estimates align directionally with IVW but show marginally wider confidence intervals, consistent with its pleiotropy-robust design. (Fig. 9D). Leave-one-out analysis demonstrated that systematically conducting MR analysis on the remaining SNPs after removing each SNP produced consistent results (Fig. 8C), indicating the robustness of this finding. Cochran *Q* test did not reveal any heterogeneity in IL-6 results (*Q* = 1.52, *P* = .91). MR-Egger regression and MR-PRESSO analyses indicated the absence of horizontal pleiotropy in the IL-6 results (*P* = .47).

## 4. Discussion

As mentioned earlier, the 13 herbs in this formula contain at least 171 active ingredients. Among the top 5 active ingredients and core targets screened by the topological parameters of network pharmacology, quercetin possesses potent anti-inflammatory properties and can reduce the inflammatory response. Studies have shown that quercetin can inhibit the production of several inflammatory factors, such as TNF-α and IL-1β, thereby alleviating the symptoms of inflammatory diseases,^[27]^ which is in line with the results of our network pharmacology screening, where quercetin and TNF-α were both the highest degree values for the treatment of moderate to severe plaque psoriasis biologically molecularly targeted drugs also include TNF inhibitors.^[5]^ Dihydrobaicalin has significant anti-inflammatory and antioxidant properties, and studies have demonstrated its ability to mitigate atherosclerosis by inhibiting the NF-kappaB and p38 MAPK signaling pathways.^[28]^ In addition, baicalein has shown some potential in anticancer studies. For example, it can inhibit the growth of human osteosarcoma cells and induce apoptosis through a mechanism that may be related to the inhibition of the AKT signaling pathway^[29]^ which is also included in our selection of ATK1. Kinobeon A, a compound extracted from the medicinal plant Bitter Ginseng (safflower), exhibits remarkable tyrosinase inhibition. According to the study, Kinobeon A showed potent tyrosinase inhibition in an in vitro assay,^[30]^ tyrosinase is a key enzyme in melanin synthesis and its activity directly affects skin pigmentation. Tyrosinase activity may be disturbed in patients with psoriasis, resulting in an abnormal pigment metabolism. For example, it has been shown that tyrosinase activity may be reduced in the skin of patients with psoriasis, which may be related to the pathogenesis of psoriasis.^[31]^ Some studies have suggested that pyrethrin and its derivatives may have potential anticancer effects. For example, 1 study found that pyrethrin inhibited the activity of an enzyme called AMPD2 in lung cancer cells and promoted its degradation.^[32]^ In conclusion, the 5 active ingredients of XBT that we screened not only have anti-inflammatory effects, but also possess some anticancer effects in psoriasis patients, as the long-term development of the disease can increase the risk of cardiovascular diseases^[33–36]^ and tumors,^[37]^ especially IL-17, which plays an important role in the pathological mechanisms of psoriasis, may be a key molecule linking psoriasis and cardiovascular disease risk,^[38]^ and IL-17 is included in the target mechanism of action of XBT, and biologically targeted drugs for the treatment of psoriasis may also increase the risk of tumors. As for the potential effects of XBT on the inflammatory process, as well as multiple links and pathways in the process of tumor formation, XBT may also reduce cardiovascular disease and cancer risks in psoriasis patients, which may further improve the prognosis of patients with psoriasis.

The High Degree Value Core Targets of the XBT TNF are pro-inflammatory cytokines, and TNF exacerbates the inflammatory response by activating the NF-kB signaling pathway, which regulates the expression of a variety of inflammation-associated genes, such as IL-1, IL-6 (also High Degree Value Core Targets), and IL-8.^[39]^ TNF-α activates dendritic cells through TNFRI/II receptors, promotes the secretion of IL-23, drives Th17 cell polarization, and enhances the pro-inflammatory effect of IL-17A on keratinocytes (KCs).^[40]^ TNF inhibitors such as infliximab, adalimumab, and etanercept are the preferred biologics for the treatment of moderate to severe psoriasis. By blocking TNF-α activity, they can significantly improve patients’ symptoms and inhibit the inflammatory response at the same time.^[41]^ GAPDH plays an important role in the modulation of the inflammatory response, especially by binding to the mRNA of pro-inflammatory factors such as TNF-α and IL-6. Under normal conditions, GAPDH can inhibit the translation of these mRNAs by binding to their 3’ untranslated regions, thereby reducing the expression of pro-inflammatory factors,^[42]^ however, it has been shown that high glucose induces an increase in the expression of inflammatory factors such as TNF-α and IL-6.^[43,44]^ In addition, high glucose levels lead to increased oxidative stress, which further promotes an inflammatory response. In this case, the inhibitory effect of GAPDH may be disrupted, leading to the overexpression of pro-inflammatory factors.^[45]^ It has been shown that AKT1 is overactivated in psoriatic skin, especially in keratin-forming cells (e.g. HaCaT cells), where its activity is significantly increased. This hyperactivation promotes keratinocyte proliferation mainly by phosphorylating downstream substrates such as FOXO. The PI3K/Akt pathway is considered an important mechanism in the development and progression of psoriasis.^[46]^ An increasing amount of evidence indicates that activation of this pathway by external or internal stimuli can lead to epidermal hyperplasia, immunopathology, angiogenesis, and other physiological or pathological processes related to psoriasis.^[47]^ The latest research has found that AKT1 phosphorylation activates mTORC1, promotes KC proliferation and inhibits apoptosis, and its activity is 3 times higher in psoriatic lesions than in normal skin.^[41]^ IL-6 enhances the nuclear translocation efficiency of STAT3 through AKT1, while STAT3 can feedback activate PI3K, forming a positive feedback for the proliferation-promoting signal^.[48]^ Pharmacological interventions targeting AKT1 and its associated pathways have been shown to be effective in modulating the pathological process of psoriasis. For example, AKT inhibitors reduce cell proliferation and inflammatory responses in psoriatic skin.^[49,50]^ IL1B expression levels are significantly elevated in patients with psoriasis and are strongly correlated with disease severity, which could indicate that AKT1 is a potential target for psoriasis treatment. IL1B expression is increased in psoriatic lesions, especially in keratinocytes, where its activity is enhanced, leading to an increased inflammatory response.^[51]^ In addition, IL1B exacerbates the inflammatory process in psoriasis by promoting the generation and maturation of Th17 cells.^[52]^ IL1B is also involved in the immunopathological mechanisms underlying psoriasis. For example, IL1B activates the NLRP3 inflammasome^[51]^ which promotes the secretion of inflammatory factors such as IL-17 and IL-23, which play a key role in the inflammatory response in psoriasis. Meanwhile, IL1B enhances the activation of the NF-κB signaling pathway through a positive feedback mechanism, further amplifying the inflammatory response. Notably, genetic polymorphisms in IL-6 are associated with the risk of psoriasis. It was found that certain polymorphisms in the IL-6 gene (e.g., −174 G > C) may increase the risk of developing psoriasis,^[53]^ which is consistent with our MR results. However, IL-6 is not a specific marker for psoriasis as its aberrant expression may be associated with other diseases. Recent studies have found that IL-6 drives psoriasis inflammation and abnormal proliferation of KCs through multiple signaling pathways. Serum IL-6 concentration in psoriasis patients is positively correlated with blood sedimentation, lesion area, and PASI index of psoriasis patients, and psoriasis patients with high levels of IL-6 are more prone to joint damage, which can be used as an indicator of psoriasis inflammatory activity, and there is a synergistic effect between IL-6 and AKT1. In addition, IL-6 has a synergistic effect with AKT1, IL-6 enhances the transcriptional activity of STAT3 by activating AKT1, and induces the high expression of LIM structural domain protein 4, which leads to uncontrolled proliferation and abnormal differentiation of KCs.^[54]^ Clinical studies have shown that IL-6 antibody has a good therapeutic effect on a mouse model of psoriasis, and the mechanism may be related to the effect on Th17/Th22 and dendritic cell levels through inhibition of the IL-6/STAT3 pathway.^[55]^ Based on the enrichment analysis of the signaling pathways in the results, we can see that some of the ingredients contained in XBT can act on 1 or several links in the inflammatory signal transduction mechanism, showing the wide-ranging effects of XBT. First of all, we can see that some of the ingredients contained in XBT have a close connection with the interleukin-mediated inflammatory response mechanism, and these pathway diagrams show that several members of the interleukin family play important roles in inflammatory signal transduction and are also important factors in the pathogenesis of psoriasis, or the target of the action of the ingredients contained in XBT, and we have also seen in the signaling network that, the target of the ingredients of XBT We also see in the signaling network that the targets of the ingredients of the XBT also contain molecular mechanisms related to atherosclerotic plaques, and this result suggests that the XBT may have a role in the prevention and treatment of psoriasis-associated cardio-cerebrovascular diseases. Several Chinese herbs such as jinyinhua, dihuang, and huangqin have been shown to be effective in treating these diseases. Therefore, the use of XBT for psoriasis can not only effectively intervene to inhibit the inflammatory response, but also prevent and control cardiovascular diseases to achieve the effect of both symptoms and root causes. Studies indicate psoriasis patients face elevated risks of myocardial infarction and stroke,^[3,56,57]^ suggesting XBT may confer additional cardiovascular benefits.

In conclusion, XBT has more components, and network pharmacology reveals that its therapeutic action not only controls psoriasis by eliminating inflammation, but also possesses a wide range of therapeutic effects, such as anti-tumor effects and treatment of cardiovascular diseases. In this study, we explored the multifaceted mechanism of XBT in the treatment of psoriasis from the perspective of drug intervention in the disease using the target as a nexus, which was validated by molecular docking and MR analysis. However, validation through additional experiments is required, given our reliance on public database-derived drug component/target/patient data. Overall, our study provides valuable insights for clinical and future research on the therapeutic effects of XBT in psoriasis.

The multi-target mechanisms of XBT revealed in this study suggest its potential as a complementary or alternative therapy for psoriasis. Compared to conventional biologics (e.g., TNF-α inhibitors and IL-17/IL-23 antagonists), which are costly, require long-term injections, and may increase infection or tumor risks.^[58,59]^ XBT’s natural components might offer a safer profile while maintaining anti-inflammatory efficacy. For instance, the inhibition of TNF-α, IL-6, and AKT1 pathways by XBT aligns with the action of biologics, yet its multi-component nature may reduce drug resistance and systemic toxicity. Additionally, XBT’s potential benefits in mitigating cardiovascular risks could address comorbidities common in psoriasis patients, a limitation of current targeted therapies. However, this study’s reliance on public databases introduces limitations, such as incomplete patient-specific data and unvalidated molecular interactions.^[58]^ Future research should prioritize prospective clinical trials to verify XBT’s efficacy and safety, particularly its long-term impact on psoriasis-associated cardiovascular and neoplastic risks. Experimental designs incorporating multi-omics approaches and real-world evidence are recommended to validate the network pharmacology predictions. Furthermore, optimizing XBT formulations could enhance bioavailability and therapeutic precision, and future research should focus on more high-quality randomized controlled trials to further investigate this topic.^[60]^

This study revealed the multi-target mechanisms of XBT in treating psoriasis and provided genetic evidence supporting its efficacy. The integration of network pharmacology and MR analysis offers a novel approach for evaluating XBT formulas and identifying therapeutic targets in complex diseases. Network pharmacology and MR analysis offer novel approaches for evaluating XBT formulas and identifying therapeutic targets for complex diseases.

## Acknowledgments

The authors extend their profound gratitude to the dedicated researchers and participants of the study for their invaluable contributions.

## Author contributions

**Data curation:** Jie Wang.

**Funding acquisition:** Changhui Wen.

**Investigation:** Wentao Hu, Chunlan Wu.

**Methodology:** Wentao Hu.

**Project administration:** Wentao Hu.

**Resources:** Yifang Jiang.

**Supervision:** Wentao Hu, Changhui Wen.

**Software:** Min Jia, Chunlan Wu.

**Validation:** Chunlan Wu.

**Visualization:** Chunlan Wu.

**Writing – original draft:** Wentao Hu.

**Writing – review & editing:** Wentao Hu.

## Supplementary Material


